# Cottonseed protein, oil, and mineral status in near-isogenic *Gossypium hirsutum* cotton lines expressing fuzzy/linted and fuzzless/linted seed phenotypes under field conditions

**DOI:** 10.3389/fpls.2015.00137

**Published:** 2015-03-19

**Authors:** Nacer Bellaloui, Salliana R. Stetina, Rickie B. Turley

**Affiliations:** Crop Genetics Research Unit, Plant Physiology, United States Department of Agriculture, Agricultural Research ServiceStoneville, MS, USA

**Keywords:** cottonseed protein, cottonseed oil, cottonseed composition, minerals, near-isogenic cotton, cotton mutants

## Abstract

Cotton is an important crop in the world and is a major source of oil for human consumption and cotton meal for livestock. Cottonseed nutrition (seed composition: protein, oil, and minerals) determines the quality of seeds. Therefore, maintaining optimum levels of cottonseed nutrition is critical. Physiological and genetic mechanisms controlling the levels of these constituents in cottonseed are still largely unknown. Our previous research conducted under greenhouse conditions showed that seed and leaf nutrition differed between fuzzless and fuzzy seed isolines. Therefore, the objective of this research was to investigate the seed fuzz phenotype (trait) effects on seed protein, oil, N, C, S, and minerals in five sets of near-isogenic mutant cotton lines for seed fuzz in a 2-year experiment under field condition to evaluate the stability of the effect of the trait on seed nutrition. The isolines (genotypes) in each set differ for the seed fuzz trait (fuzzless/linted seed line, *N* lines, and fuzzy/linted seed line, *F* lines). Results showed that seed protein was higher in the fuzzy genotype in all sets, but seed oil was higher in fuzzless genotype in all sets. The concentrations of seed Ca and C were higher in all fuzzless genotypes, but N, S, B, Fe, and Zn were higher in most of the fuzzy genotypes. Generally, minerals were higher in leaves of *F* lines, suggesting the translocation of minerals from leaves to seeds was limited. The research demonstrated that fiber development could be involved in cottonseed composition. This may be due to the involvement of fiber development in carbon and nitrogen metabolism, and the mobility of nutrients from leaves (source) to seed (sink). This information is beneficial to breeders to consider fuzzless cottonseed for potential protein and oil use and select for higher oil or higher protein content, and to physiologists to further understand the mobility of minerals to increase the quality of cottonseed nutrition for food and feed.

## Introduction

Cotton is a major crop in the world (Yu et al., 2012). Cotton fiber is a source of natural textile, and cottonseed is a source of oil for human consumption, cotton meal and minerals for livestock feed (Yu et al., 2012; He et al., 2013). Therefore, maintaining high quality fiber and cottonseed nutritional value is critical. There are four cultivated cotton species, *Gossypium hirsutum* (about 95% of the cultivated cotton), *G. barbadense*, *G. arboreum*, and *G. herbaceum* together (the last three represent about 5%) (Turley et al., 2007; Padmalatha et al., 2012; Bellaloui and Turley, 2013; Stetina et al., 2014). Cotton fibers are developed from the ovule epidermis and are single-celled seed trichomes, and about 30% of the seed epidermal cells differentiate into spinable fibers (Arpat et al., 2004; Wilkins and Arpat, 2005). The development of cotton fibers comprises four stages named fiber cell initiation, fiber cell elongation/primary cell wall (occurs up to 20 days post-anthesis), fiber cell synthesis/secondary cell wall (occurs between 5 and 15 days post-anthesis, and the secondary cell wall synthesis begins at about 20 days post-anthesis and reaches up to 45 days post-anthesis), and maturation (occurs from 45 to 50 days post-anthesis) where fibers dehydrate and produce mature cotton lint (Ji et al., 2003; Arpat et al., 2004; Wilkins and Arpat, 2005).

Upland cotton (*Gossypium hirsutum*) is the predominant species commercially cultivated in the United States and produces two types of fiber on cottonseed, lint and fuzz. Lint fiber is economically valuable, and it is longer and develops faster than the fuzz (Stewart, 1975; Seagull and Giavalis, 2004; Stetina et al., 2014). During ginning process, lint fiber is removed and fuzz fiber left behind (Bechere et al., 2009). There are two naturally occurring mutations in cotton allowing the development of lint fiber, but preventing development of fuzz fiber, and these are referred to as the fuzzless seed alleles and designated as genotypes *N*_1__(dominant fuzzless allele) or *n*_2_n_2_ (recessive fuzzless allele) (Turley and Kloth, 2002; Turley et al., 2007; Stetina et al., 2014).

It is reported that the fuzzless trait in cottonseed is a novel tool to use to understand the biology, genetics, and biochemical and metabolic processes (Turley et al., 2007; Padmalatha et al., 2012). In spite of the few reports available on the effects of fuzzless trait on the biology, genetics, and molecular biology (Padmalatha et al., 2012), very limited information is available on the effects of fuzzless trait on cottonseed composition (Yu et al., 2012; Bellaloui and Turley, 2013; He et al., 2013). For example, the effect of fuzzless trait was investigated on genetic products (Turley and Kloth, 2002; Gou et al., 2007; Turley et al., 2007; Zhao et al., 2009; Pang et al., 2010a,b; Liu et al., 2012), physiological (Ruan et al., 2003; Shi et al., 2006; Yang et al., 2006; Wang et al., 2010; Wang and Ruan, 2010; Zhang et al., 2011), and molecular (Machado et al., 2009; Guan et al., 2011; Walford et al., 2011). It was also reported that carbohydrate and energy metabolisms are involved in fiber development and carbon skeletons for the synthesis of cell wall polysaccharides and fatty acids (Gou et al., 2007; Yang et al., 2008; Pang et al., 2010a,b), and actin cytoskeleton to trigger the secondary cell wall synthesis (Li et al., 2002, 2005; Wang et al., 2010; Wang and Ruan, 2010).

Cottonseed quality is also determined by its content of mineral and non-mineral nutrients such as N, C, S, K, Ca, Zn, and Fe because of their direct or indirect contribution to: protein synthesis (such as N, K, S); oil (such as C and N); carbohydrates (such as C, K, B); metabolite synthesis (Cu, Zn, Fe, Mg); integrity of cell membrane and cell wall structure (Ca and B); cell membrane, lipid synthesis, energy transfer and phosphorylation reactions, carbohydrate metabolism, and nutrient active uptake processes (P); and osmoregulation, stomatal closure, carbohydrate movement, and nutrient mobility (K). The physiological and biochemical roles of these nutrients in plant growth and development were previously reported (Mengel and Kirkby, 1982; Marschner, 2012). The benefit of minerals to human health was also previously reported. For example, an unbalanced diet of micronutrients such as Fe, Zn, leads to human malnutrition (Samman et al., 1998; Fletcher et al., 2004; Lu et al., 2008). Therefore, maintaining an optimum level of Fe, Zn, Cu, Mo, Mn (Zhang et al., 2004; Heinemann et al., 2005) in seeds of major crops that are used for protein and oil sources is critical. It was reported that nutrient uptake, translocation, redistribution, and accumulation are processes controlling the concentrations of minerals in seeds (Grusak and DellaPenna, 1999; White and Broadley, 2005), and most of the genetic basis of these process are not known (Ding et al., 2010).

To our knowledge this is the first comprehensive report to use near-isogenic cotton mutant genotypes for the fuzzless trait to evaluate its effects on cottonseed nutrition (protein, oil, N, S, C, and minerals) under field conditions. Our hypothesis was that because several metabolic processes including carbon metabolism and polysaccharide cell wall synthesis are involved in fiber development (Li et al., 2002, 2005; Gou et al., 2007; Yang et al., 2008; Wang et al., 2010; Wang and Ruan, 2010; Pang et al., 2010a,b), cottonseed nutrition will be influenced as protein and oil are results of carbon, nitrogen, and carbohydrate metabolism. Since we observed differences in leaf and seed nutrients between lines, we hypothesized that nutrients in lint fiber may be impacted as well. Therefore, we investigated the effect of fuzz trait on nutrient content in lint for 1 year only to obtain preliminary results for future research planning.

## Materials and methods

### Germplasm development

The development of near-isogenic lines was described in detail elsewhere (Stetina et al., 2014). The near-isogenic lines (NILs), one expressing the fuzzy/linted and one expressing fuzzless/linted phenotype were developed in five upland cotton backgrounds (Stetina et al., 2014). The following are five sets of *F* and *N* lines/genotypes: Sure-Grow (SG) 747 *F* vs. SG 747 *N*; MD51*ne F* vs. MD51*ne N*; STV 7A*gl F* vs. STV 7A*gl N*; DP 5690 *F* vs. DP 5690 *N*; DES 119 *F* vs. DES 119 *N*. Genotype SA 243 (“Ballard naked seed,” PI 528610) was the fuzzless parent with the dominant fuzzless seed allele, *N_1_N_1_* (Turley et al., 2007), and DP 444 BG/RR (Monsanto Company, St. Louis, MO, USA) was used as the commercial check.

### Field management and growth conditions

A field experiment was conducted in 2012 and 2013 at Stoneville, MS, USA. Field management and growth conditions were previously detailed in Stetina et al. (2014). Briefly, cottonseeds were planted on four-row plots spaced 1.02 m apart and each plot was 9.14 m long with a 3.04 m alley between plots. Planting dates were 25 April 2012 and 13 May 2013. Insecticides and fungicides were applied to control pest and diseases. Field management was conducted according to the standard agronomic practices for cotton production in the Mississippi Delta region (http://msucares.com/crops/cotton/index.html). Cotton bolls were harvested from 10 adjacent plants at three (2012) and four (2013) intervals to prevent the loss of fuzzless cottonseed content due to rain and wind. The harvest intervals were on 28 August, 6 September, and 27 September in 2012, and on 5 September, 12 September, 17 September, and 27 September in 2013. A defoliant was used prior to the last harvest date each year. Cottonseed samples were processed at the USDA ARS Cotton Ginning Laboratory at Stoneville, MS, USA and saw-ginned on 20 November 2012 and 12 November 2013. Cottonseed were collected and acid-delinted for seed composition analyses. Soil samples were taken across the field by dividing the field into four main sections and taking 20–25 samples from each section at depth of 30.5 cm. Therefore, one composite sample (combined 20–25 samples) represented each section. Analysis of the four samples, representing the four sections, showed uniformity in main soil nutrients. Therefore, the soil nutrients shown in here are average of the four sections representing the entire field. For leaf sampling, the most recent fully expanded leaves were taken from each plot. Fourteen leaves were taken from the middle two rows (7 leaves were taken along each one middle row to ensure equal distribution of leaves along each row). Leaves were oven-dried at 65°C and ground into fine particle with Laboratory Mill 3600 (Perten, Springfield, IL). Seed and leaf C, N, K, Ca, Mn, and Zn were analyzed at the University of Georgia's Soil, Plant, and Water Laboratory, Athens, GA. Seed protein, oil, B, Fe, P, and leaf B, Fe, and P were analyzed as described below.

### Boron analysis

Boron concentrations in the most recently fully expanded leaves at boll stage and mature seeds were determined according to Lohse (1982) using the azomethine-H method, and samples were prepared according to John et al. (1975). Boron concentration was determined spectrophotometrically by reading the samples at 420 nm using a Beckman Coulter DU 800 spectrophotometer (Fullerton, CA). The concentration of B was measured after color development, and B concentration was expressed as mg B kg^−1^ dwt.

### Iron analysis

Iron concentrations in the most recently fully expanded leaves at boll stage and mature seeds were measured according to Bandemer and Schaible (1944) and Loeppert and Inskeep (1996). The concentration was determined by acid wet digestion (Analytical Methods Committee, 1959), extraction, and reaction of the reduced ferrous Fe using 10 ml of 0.02 M 1,10-phenanthroline, and the samples were prepared for measurement using a quinol solution of 1% (w/v) reagent. Concentrations of Fe in leaves and seeds were measured spectrophotometrically at 510 nm using a Beckman Coulter DU 800 spectrophotometer (Fullerton, CA).iron was expressed as mg Fe kg^−1^ dwt.

### Phosphorus analysis

Phosphorus concentrations in leaves and mature seeds were determined according to Cavell (1955) using the yellow phosphor-vanado-molybdate complex. Briefly, 2 g of dried ground samples were ashed and 10 ml of 6 M HCl was added. The samples, then were placed in a water bath to evaporate the solution to dryness. Then, a 2 ml of 36% v/v HCl was added and the samples were boiled. Ten milliliters of distilled water was added to the samples and the solution was brought to boil for a few seconds, and then diluted to 50 ml with distilled water, and then filtered. To measure P concentration, a reagent of 5 ml of 5 M HCl and 5 ml of ammonium molybdate–ammonium metavanadate was freshly prepared and added to the filtrate. The concentration of P was measured spectrophotometrically using a Beckman Coulter DU 800 spectrophotometer at 400 nm. The concentration of P in the sample was expressed as a percentage.

### Analyses of N, S, C in leaves and seed

The most recently fully expanded leaves during boll stage and mature seeds were analyzed for minerals, N, S, and C concentrations by digesting 0.6 g of dried, ground seed in HNO_3_ in a microwave digestion system. The concentrations of minerals in the samples were determined using inductively coupled plasma spectrometry (ICP) (Bellaloui and Turley, 2013; Bellaloui et al., 2014). For N, S, and C measurements, a 0.25 g ground-dried sample was combusted in an oxygen atmosphere at 1350°C to convert elemental N, S, and N, S, and C were measured by an elemental analyzer using thermal conductivity cells (LECOCNS-2000 Elemental Analyzer, LECO Corporation, St. Joseph, MI) (Bellaloui et al., 2011, 2014).

### Analyses of minerals, N, S, and C in soil

Mineral concentrations in soil, N, S, and C were analyzed at The University of Georgia's Soil, Plant, and Water Laboratory, Athens, GA. The concentrations of the minerals K and Mn were determined using a 5-g soil: 20 ml Mehlich-1 solution and analyzed using inductively coupled plasma (ICP) spectrometry (Bellaloui et al., 2009). Percentages of N, S, and C were determined in a 0.25-g sample of soil by combusting samples in an oxygen atmosphere at 1350°C, and converting elemental N, S, and C into N_2_, SO_2_, and CO_2_ gasses, and N, S, and C were determined by elemental analyzer using thermal conductivity cells (LECOCNS-2000 Elemental Analyzer LECO Corporation, St. Joseph, MI, USA) (Bellaloui et al., 2009). Concentrations of N, S, and C were expressed as percentages.

### Cottonseed protein and oil analysis

Mature cottonseeds were collected from each plot and analyzed for protein and oil. Briefly, approximately 25 g of seed was ground using a Laboratory Mill 3600 (Perten, Springfield, IL). Protein and oil in cottonseed were analyzed by near infrared reflectance according to Wilcox and Shibles (2001) and Bellaloui and Turley (2013) using a diode array feed analyzer AD 7200 (Perten, Springfield, IL). Calibrations were developed using Perten's Thermo Galactic Grams PLS IQ software, and the calibration equation was established according to AOAC methods (Association of Official Analytical Chemists (AOAC), 1990a,b). Cottonseed protein and oil were expressed on a seed dry matter basis (Wilcox and Shibles, 2001; Boydak et al., 2002; Bellaloui and Turley, 2013).

### Experimental design and data analysis

This experiment was a part of a large experiment that was designed in a split-plot. The main plot was line (genotype), and plots were arranged in a randomized complete block design with 4 replications. The subplot was ginning method (Stetina et al., 2014). In the current study, we were interested in the conventional saw ginning method only. Therefore, the only factors under the study were line, year, and their interactions. Analysis of variance was conducted using Proc Mixed model in SAS (Statistical Analysis System; SAS Institute, Inc., Cary, NC, 2002-2012). Year (Y), Line, and Y × Line interactions were modeled as fixed effects, and replicates and their interactions were considered random effects. Means were separated by Fisher's protected least significant difference test at the 5% level of significance using SAS (2002–2012). Correlation was performed using PROC CORR in SAS (2002–2012).

## Results and discussion

### Analysis of variance

ANOVA showed that year and line were the major sources of variability for seed composition constituents (Table 1). Based on *F*-value, year is more important for some constituents such as P, Mg, and B than line, suggesting that growing season may have also an influence on these constituents. For others constituents such as protein, N, C, and Zn, genotypic effects were more important than year effects, suggesting the significant effects of genetic factors in controlling the accumulation of these constituents. There were no significant effects of year on oil, S, K, and Mn, suggesting that the response of these constituents was similar in each year. All constituents were influenced by line, but the magnitude of the accumulation in seeds differed, depending on the line. For leaf N, S, and minerals (Table 2), year was significant for all nutrients, except for Fe and Mn. Line had significant effects for all nutrients, except for P and K, suggesting that the levels of nutrients in leaves were significantly influenced by the line. Since the *F*-value of year × line interactions was smaller (less significant: its contribution to the model was smaller) compared with that of the main effect of year or line, the data were combined across the 2 years.

**Table 1:** **Effect of source of variance (F- and P-values) on cottonseed protein concentration (g kg^−1^), oil concentration (g kg^−1^), N, C, S, P, K, Ca, Mg percentages (%), and Fe, B, Mn, and Zn concentration (mg kg^−1^)**.

**Effect**	**Minerals**																									
	**Protein**		**Oil**		**N**		**C**		**S**		**P**		**K**		**Ca**		**Mg**		**Fe**		**B**		**Mn**		**Zn**	
	**F**	**P**	**F**	**P**	**F**	**P**	**F**	**P**	**F**	**P**	**F**	**P**	**F**	**P**	**F**	**P**	**F**	**P**	**F**	**P**	**F**	**P**	**F**	**P**	**F**	**P**
Year	15.8	***	1.4	NS	15.8	**	8.2	*	3.3	NS	42.8	***	0.4	NS	62.0	**	44.3	***	16.9	**	36.7	**	1.0	NS	2.3	NS
Line	20.4	***	13.4	***	20.4	***	17.3	***	5.0	***	5.9	***	4.3	***	62.6	***	8.9	**	16.1	***	24.5	***	5.1	***	59.3	***
Year × Line	2.3	**	3.6	***	2.3	**	3.9	***	1.4	NS	1.8	*	1.5	NS	1.6	NS	2.2	*	3.5	**	1.4	NS	0.4	NS	13.0	***

*Cotton was grown in the field in 2012 and 2013 in Stoneville, MS, USA*.

* Significance at P ≤ 0.05;

** significance at P ≤ 0.01;

*** *significance at P ≤ 0.001*.

**Table 2:** **Effect of source of variance (F- and P-values) on leaf macro-nutrients (%) and micro-nutrients (mg kg^−1^)**.

**Effect**	**Macro-nutrients**												**Micro-nutrients**							
	**N**		**S**		**P**		**K**		**Ca**		**Mg**		**Fe**		**B**		**Mn**		**Zn**	
	**F**	**P**	**F**	**P**	**F**	**P**	**F**	**P**	**F**	**P**	**F**	**P**	**F**	**P**	**F**	**P**	**F**	**P**	**F**	**P**
Year	18.1	**	87.7	***	11.6	***	6.2	*	51.1	***	102.3	***	1.9	NS	85.0	***	1.6	NS	25.0	***
Line	15.5	***	32.5	***	43.5	NS	32.3	NS	12.7	***	53.2	***	55.1	***	21.3	***	4.1	***	10.8	***
Year × Line	2.6	**	3.7	***	1.7	NS	1.9	*	0.6	NS	5.4	***	3.9	***	4.4	***	2.4	**	4.1	***

*Cotton was grown in the field in 2012 and 2013 in Stoneville, MS, USA*.

* Significance at P ≤ 0.05;

** significance at P ≤ 0.01; significance at

*** *P ≤ 0.001*.

### Effects of fuzz trait on seed and leaf composition

**Figure 1:**
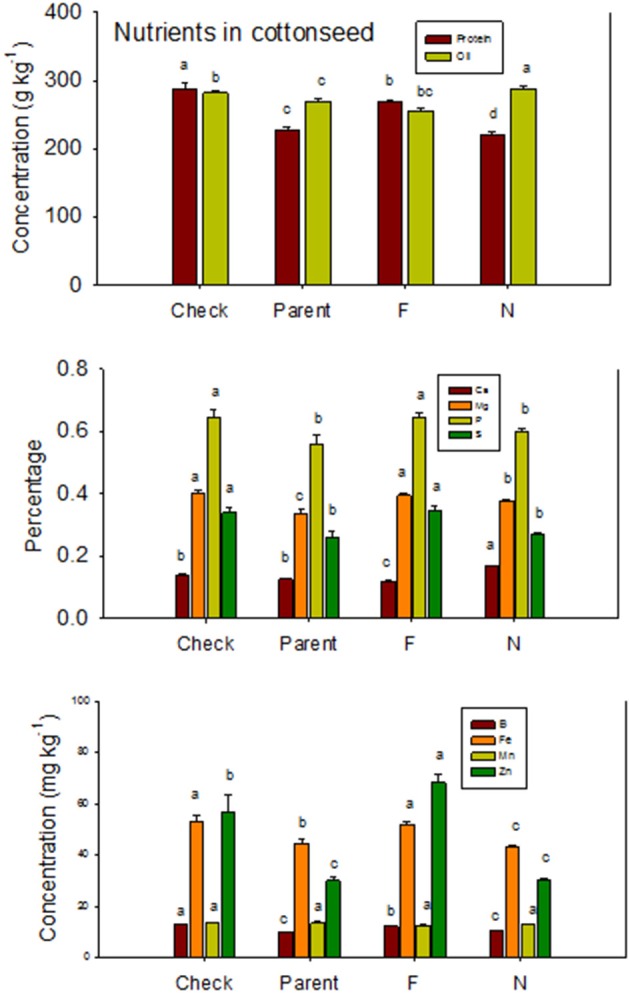
**Effects of cottonseed fuzz trait on cottonseed protein, oil, and nutrients in near-isogenic *Gossypium hirsutum* lines expressing fuzzy/linted (*F*) and fuzzless/linted (*N*) seed phenotypes, the fuzzless/linted parent SA 243 (parent), and the fuzzy/linted commercial cultivar DP 444 BG/RR (check)**. Cotton was grown in the field in 2012 and 2013 in Stoneville, MS, USA. Bars show means ± standard error of the mean (SE). Within each constituent, means with the same letter are not significantly different at 5% as determined by Fisher's LSD test.

**Figure 2:**
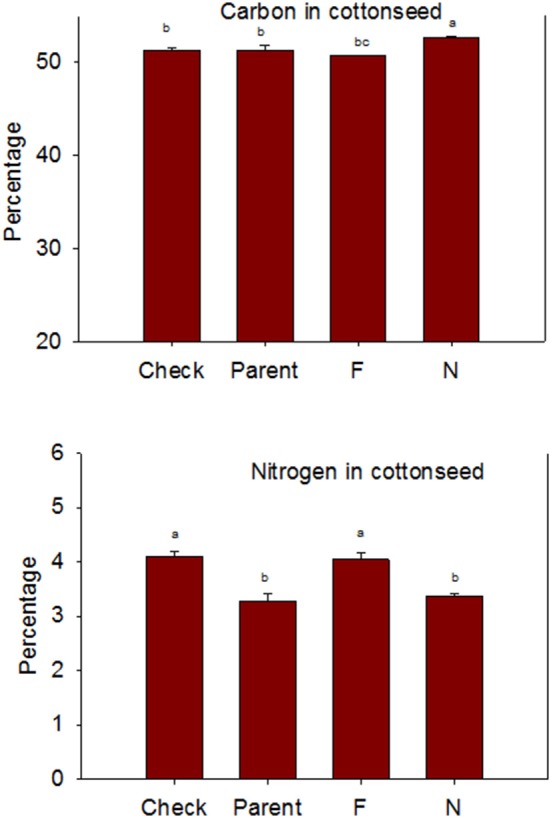
**Effects of cottonseed fuzz trait on cottonseed carbon (C) and nitrogen (N) in near-isogenic *Gossypium hirsutum* lines expressing fuzzy/linted (*F*) and fuzzless/linted (*N*) seed phenotypes, the fuzzless/linted parent SA 243 (parent), and the fuzzy/linted commercial cultivar DP 444 BG/RR (check)**. Cotton was grown in the field in 2012 and 2013 in Stoneville, MS, USA. Bars show means ± standard error of the mean (SE). Within each constituent, means with the same letter are not significantly different at 5% as determined by Fisher's LSD test.

Lines with fuzzy seeds (*F*) had higher concentrations of seed protein, N and S than lines with fuzzless seeds (*N*) (with exception of STV 7A*gl N*), but *N* lines had higher oil and C than in seeds of *F* lines (Table 3). Calcium levels in seed were higher in *N* lines than in *F* lines. Minerals K, Mg, P, and Zn in seeds were higher in *F* lines than in *N* lines. Nitrogen (for STV 7A*gl*) and Mn (for DP 5690, SG 747, and STV 7A*gl*) did not follow the general trend of other nutrients of higher nutrient accumulation in *F* lines. Instead *N* did not differ between *N* and *F* lines in STV 7A*gl*, and Mn was higher in the *N* line for DP 5690, but did not differ between *N* and *F* lines in SG 747 and STV 7A*gl*. Except for Mn in DES 119, MD 51*ne*, and STV 7A*gl*, and Ca, P, and N for DP 5690, N and S and all minerals in leaves were higher in *N* lines than in *F* lines (Table 4). When the data were expressed across all lines for the fuzz trait (*F* vs. *N*) (Figures 1, 2), oil, C, and Ca, were higher in cottonseed in *N* lines than in *F* lines, but protein and other minerals, except Mn, were higher in *F* lines than in *N* lines, supporting data expressed on line set basis. Fuzzless and fuzzy lines had similar concentrations of Mn.

**Table 3:** **Effect of fuzzless/linted (*N*) and fuzzy/linted (*F*) cottonseed phenotypes on cottonseed protein concentration (g kg^−1^), oil concentration (g kg^−1^), N, C, S, P, K, Ca, Mg percentages (%), and Fe, B, Mn, and Zn concentration (mg kg^−1^)**.

**Line/genotype**	**Protein**	**Oil**	**C**	**N**	**S**	**Ca**	**K**	**Mg**	**P**	**B**	**Fe**	**Mn**	**Zn**
SA 243	226 ef	270 ef	51.3 d	3.27 g	0.26 e	0.13 f	0.94 e	0.34 g	0.56 g	10.0 f	44.3 e	13.4 c	29.9 e
DP 444 BG/RR	287a	282 cd	51.3 d	4.10 bc	0.34 c	0.14 e	1.03 bc	0.40 b	0.64 bc	12.8 a	52.8 b	13.3 cd	56.9 c
DES 119 *N*	240 d	293 b	52.8 b	3.48 ef	0.30 d	0.18 a	0.95 e	0.41b	0.65 b	11.7 b	49.0 c	14.0 b	33.9 e
DES 119 *F*	276 b	260 fg	50.8 e	4.32 a	0.40 a	0.12 g	1.05 b	0.43 a	0.72 a	12.8 a	62.4 a	14.6 a	81.7 a
DP 5690 *N*	217 g	323 a	53.3 a	3.25 g	0.27 e	0.15 c	0.91 f	0.35 f	0.55 g	9.3 g	39.9 g	12.2 f	25.9 f
DP 5690 *F*	252 c	275 fg	50.9 e	3.95 c	0.37 b	0.11 h	0.99 d	0.38 cd	0.59 f	11.6 b	48.7 cd	11.5 g	76.8 b
MD 51*ne N*	221 fg	274 fg	52.2 c	3.27 g	0.26 e	0.18 a	0.95 e	0.37 e	0.59 ef	10.7 d	44.6 e	13.6 c	30.8 e
MD 51*ne F*	274 b	257 gh	50.9 e	4.15 ab	0.35 bc	0.14 d	1.05 bc	0.39 c	0.62 cd	11.7 b	50.6 c	12.2 f	75.6 b
SG 747 *N*	229 e	288 bc	52.1 c	3.31 fg	0.26 e	0.17 b	0.94 e	0.37 de	0.61 de	11.0 c	42.2 f	12.9 de	31.2 e
SG 747 *F*	285 a	250 h	50.3 g	4.11 bc	0.31 d	0.12 fg	1.02 c	0.38 c	0.65 bc	12.9 a	50.1 c	12.7 e	50.7 d
STV 7A*gl N*	197 h	264 e	52.7 b	3.57 de	0.26 e	0.15 c	0.95 e	0.38 cd	0.59 ef	10.4 e	39.3 g	11.3 g	30.0 ef
STV 7A*gl F*	255 c	234 i	50.5 f	3.72 d	0.30 d	0.11 h	1.10 a	0.40 b	0.65 b	11.8 b	47.0 d	11.4 g	55.9 c

*Cotton was grown in the field in 2012 and 2013 in Stoneville, MS, USA. Means within a column followed by the same letter are not significantly different at 5% as determined by Fisher's LSD test*.

**Table 4:** **Effect of fuzzless/linted (*N*) and fuzzy/linted (*F*) cottonseed phenotypes on leaf Ca, K, Mg, P, N, and S percentages (%), and B, Fe Mn, and Zn concentration (mg kg^−1^)**.

**Line/genotype**	**Ca**	**K**	**Mg**	**P**	**N**	**S**	**B**	**Fe**	**Mn**	**Zn**
SA 243	3.39 de	2.01 f	1.09 c	0.42 g	4.23 d	0.96 e	40.9 e	78.6 g	166.6 cd	37.4 d
DP 444 BG/RR	3.36 e	1.88 g	0.95 d	0.39 h	3.84 f	0.84 f	40.2 e	77.6 g	158.3 f	37.4 cd
DES 119 *N*	4.31 b	3.48 b	1.22 b	0.75 a	4.41 c	1.61 b	51.9 b	120.2 b	170.8 b	42.4 a
DES 119 *F*	3.09 f	2.38 e	0.78 e	0.58 d	3.40 g	0.96 e	40.2 e	90.1 de	172.8 ab	30.8 e
DP 5690 *N*	3.58 cd	2.78 c	1.00 d	0.65 c	4.04 c	1.70 a	48.0 c	91.3 d	173.3 a	39.0 b
DP 5690 *F*	3.68 c	1.70 h	0.67 f	0.65 c	3.80 c	1.31 d	41.3 e	87.2 e	167.8 c	37.4 d
MD 51*ne N*	4.70 a	2.84 c	1.68 a	0.70 b	4.90 b	1.54 b	55.1 a	141.0 a	162.3 e	42.3 a
MD 51*ne F*	3.66 c	1.55 i	0.98 d	0.51 f	3.80 f	0.97 e	43.3 d	117.2 b	162.0 e	31.6 e
SG 747 *N*	4.45 b	2.61 d	1.61 a	0.64 c	4.60 b	1.42 c	50.7 b	119.7 b	165.1 d	43.5 a
SG 747 *F*	3.38 e	1.60 hi	0.93 d	0.43 g	3.81 f	0.89 ef	40.3 e	82.0 f	167.9 c	36.7 d
STV 7A*gl N*	4.88 a	3.81 a	1.22 b	0.75 a	4.81 a	1.38 cd	50.3 b	139.4 a	162.5 e	37.2 d
STV 7A*gl F*	3.40 de	2.48 e	0.75 e	0.55 e	2.98 h	0.85 f	40.2 f	107.2 c	162.5 e	38.8 bc

*Cotton was grown in the field in 2012 and 2013 in Stoneville, MS, USA*.

*Means within a column followed by the same letter are not significantly different at 5% as determined by Fisher's LSD test*.

**Figure 3:**
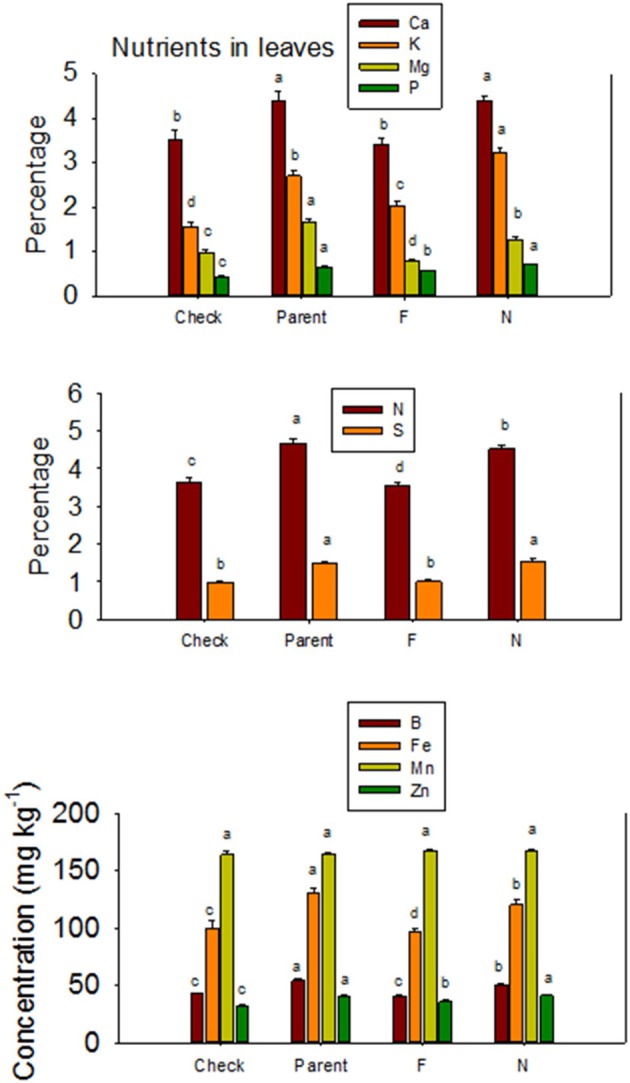
**Effects of cottonseed fuzz trait on cotton leaf nutrients in near-isogenic *Gossypium hirsutum* lines expressing fuzzy/linted (*F*) and fuzzless/linted (*N*) seed phenotypes, the fuzzless/linted parent SA 243 (parent), and the fuzzy/linted commercial cultivar DP 444 BG/RR (check)**. Cotton was grown in the field in 2012 and 2013 in Stoneville, MS, USA. Bars show means ± standard error of the mean (SE). Within each constituent, means with the same letter are not significantly different at 5% as determined by Fisher's LSD test.

The higher accumulation of nutrients of *N* lines than *F* lines in leaves was also observed when all *F* lines were compared with all *N* lines, except for Mn (Figure 3). Each line set accumulated different levels of protein, oil, or other nutrients, and the level ranged from about 197 g kg ^−1^ to 287 g kg^−1^ for protein, and 234 g kg^−1^ to 293 g kg^−1^ for oil. Similar wider ranges of other nutrients were observed between *F* and *N* lines. Cotton leaves accumulated higher concentrations of nutrients in *N* lines than in *F* lines, except for Ca and P in DP 5690, and Mn in DES 119, MD 51*ne*, and STV 7A*gl* (Table 4). Generally, both the fuzzless parent (SA 243) and the commercial cultivar DP 444 BG/RR accumulated comparable levels of nutrients to the isogenic lines sets.

**Figure 4:**
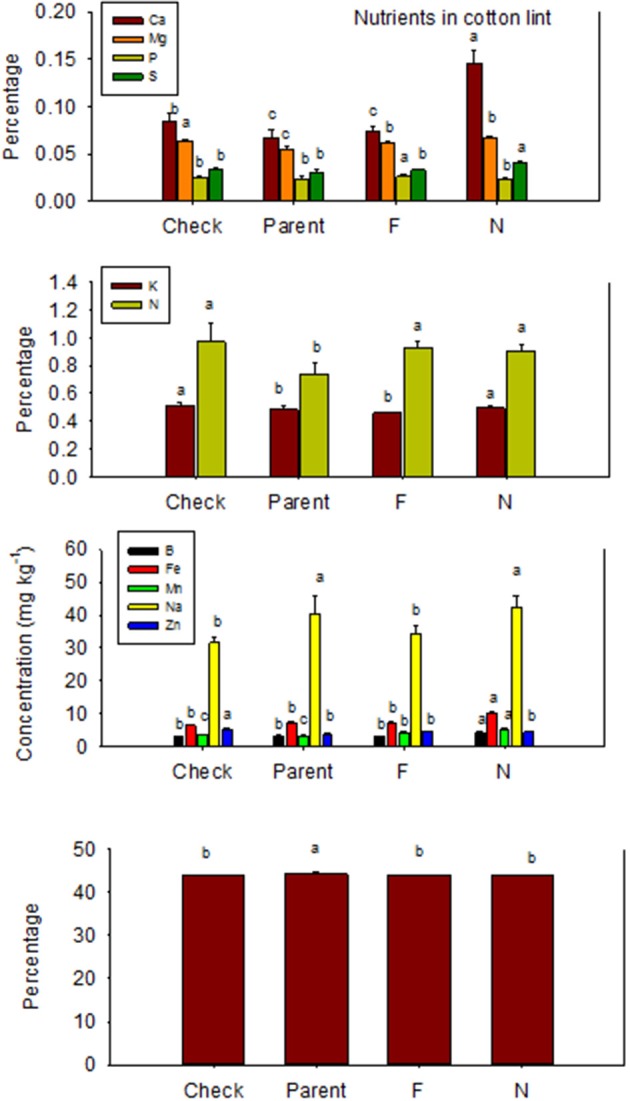
**Effects of cottonseed fuzz trait on cotton lint nutrients in near-isogenic *Gossypium hirsutum* lines expressing fuzzy/linted (*F*) and fuzzless/linted (*N*) seed phenotypes, the fuzzless/linted parent SA 243 (parent), and the fuzzy/linted commercial cultivar DP 444 BG/RR (check)**. Cotton was grown in the field in 2013 only in Stoneville, MS, USA. Bars show means ± standard error of the mean (SE). Within each constituent, means with the same letter are not significantly different at 5% as determined by Fisher's LSD test.

Nutrient accumulation in lint differed between *N* and *F* lines (Table 5). Some *N* lines accumulated more nutrients in the lint than their equivalent *F* lines, and other *N* lines accumulated less nutrients than their equivalent *F* lines, reflecting genotypic differences in nutrient accumulations in lint. For example, Ca, K, S, B, Fe, and Na contents were higher in *N* lines than in *F* lines, except in MD 51*ne* and SG 747 for K, DP 5690 and DES 119 for S, SG 747 and DP 5690 for B, DP 5690 for Fe, and MD 51*ne* for Na. All lines had at least one nutrient where *F* line was higher than *N* line, with the exception of MD 51*ne* where the *N* line was always higher or equal to the *F* line. Generally, a similar trend was also found when data were expressed on fuzz trait basis (all *F* lines vs. all *N* lines) (Figure 4).

**Table 5:** **Effect of fuzzless/linted (*N*) and fuzzy/linted (*F*) cottonseed phenotypes on cotton lint nutrients Ca, K, Mg, P, C, N, S percentages (%), and Fe, B, Mn, Na, and Zn concentration (mg kg^−1^)**.

**Line**	**Ca**	**K**	**Mg**	**P**	**C**	**N**	**S**	**B**	**Fe**	**Mn**	**Na**	**Zn**
SA 243	0.07 f	0.48 a	0.06 de	0.03 bc	44.3 a	0.74 g	0.030 e	3.1 de	7.1 ef	3.2 e	40.5 c	3.8 ef
DP 444 BG/RR	0.09 e	0.51 b	0.07 bc	0.03 bc	44.0 bc	0.97 bcd	0.033 de	3.0 e	6.4 f	3.7 ed	31.6 ef	5.3 ab
DES 119 *N*	0.15 c	0.53 a	0.07 bc	0.02 d	44.1 bc	0.88 def	0.038 bc	4.1 bc	9.6 c	5.0 bc	52.4 a	4.1 de
DES 119 *F*	0.06 f	0.46 e	0.06 c	0.03 a	43.8 de	1.05 ab	0.035 cd	2.7 e	7.0 ef	4.1 d	41.2 bc	5.5 a
DP 5690 *N*	0.11 d	0.50 b	0.07 b	0.02 cd	43.9 cde	0.79 fg	0.030 e	3.3 de	6.8 ef	4.6 c	53.0 a	3.3 f
DP 5690 *F*	0.07 ef	0.46 e	0.07 b	0.02 d	44.1 bc	0.96 bcde	0.030 e	2.9 e	8.5 d	4.8 c	45.9 b	4.1 de
MD 51*neN*	0.19 a	0.47 de	0.06 c	0.03 b	44.0 cde	0.84 f	0.053 a	4.5 b	11.1 b	6.6 a	33.2 ef	5.2 ab
MD 51*ne F*	0.09 e	0.47 cde	0.05 e	0.02 cd	44.1 bc	0.87 ef	0.038 bc	3.0 e	6.7 ef	4.7 c	34.1 de	4.5 cd
SG 747 *N*	0.11 d	0.43 f	0.06 c	0.02 d	44.0 cde	0.95 cde	0.038 bc	3.0 e	9.1 cd	3.7 ed	39.2 cd	5.2 ab
SG 747 *F*	0.09 e	0.43 f	0.06 d	0.03 b	44.0 cb	0.99 abc	0.033 de	3.6 cd	7.4 e	4.1 d	26.3 fg	4.3 cde
STV 7Agl *N*	0.17 b	0.54 a	0.08 a	0.02 cd	43.8 e	1.09 a	0.040 b	5.9 a	14.0 a	5.4 b	33.7 ef	4.5 cd
STV 7Agl *F*	0.07 ef	0.48 cde	0.07 bc	0.03 b	44.1 bc	0.79 fg	0.030 e	2.8 e	6.7 ef	3.4 e	23.6 g	4.7 bc

*Cotton was grown in the field in 2013 only in Stoneville, MS, USA*.

*Means within a column followed by the same letter are not significantly different at 5% as determined by Fisher's LSD test*.

The higher protein and lower oil concentrations in cottonseed may suggest there is a potential commercial use for fuzzless seed as a source for food (oil) and feed (cottonseed meal). Our results are in agreement with previous reports (Bechere et al., 2009). They developed homozygous naked-tufted M_8_ mutant lines and evaluated the developed lines for lint yield, fiber quality, seed oil content, ginning efficiency, and yarn spinning. They found that lint yield in the naked-tufted seed mutants was lower when compared with their original fuzzy parents. Also, they found that seed oil content in naked-tufted seed mutants was higher than fuzzy parents. Bellaloui and Turley (2013), working on similar material with the same trait, but under greenhouse conditions, and found, generally, that fuzzless cotton seeds accumulated higher oil and lower protein compared with their equivalent fuzzy seeds, showing an inverse relationship between protein and oil. The inverse relationship between protein and oil was previously reported in other species such as corn (Kebede et al., 2013), soybean (Burton, 1985; Ray et al., 2006; Bellaloui et al., 2013a,b), and cottonseed (Pettigrew and Dowd, 2011). Pettigrew and Dowd (2011) investigated the effect of irrigation and planting dates on seed oil and protein in six conventional cotton (*Gossypium hirsutum*) cultivars (DP 445BR, DP 555BR, FM 800BR, FM 960BR, ST 4892BR, and ST 5599BR), planted at late April and late May, and under irrigated and non-irrigated (dryland) field conditions in Stoneville, MS, from 2005 to 2008. They found irrigation increased the concentrations of seed oil, but decreased seed protein. However, under dryland conditions, protein was higher than oil at late April and late May planting. Their results showed opposite trend between protein and oil, agreeing with the current results.

The higher accumulation of some nutrients in seeds of *F* lines than in *N* lines, and the higher accumulation of some other nutrients in *N* lines than in *F* lines was also previously reported (Bellaloui and Turley, 2013), and this could be due to genotypic differences. The consistent higher accumulation of nutrients in leaves of *N* lines than in *F* lines across line sets suggests limited translocation of nutrients from leaves to seed, and this may explain the lower accumulation of minerals in seeds of some fuzzless isolines. For example, B showed higher accumulation in leaves and lower accumulation in seed of *N* lines than in the *F* lines. It is known that cotton has higher B requirement for growth, development, and seed quality. Although B is mobile in sugar-alcohol containing species such as rice, pears, almond, and celery, it looks like B in cotton has limited mobility (Brown and Shelp, 1997; Brown et al., 2002; Dordas, 2006) from leaves to seed. Although high concentrations of B occurred in leaves (ranged from 40 to 55 mg B kg^−1^), cottonseed contained limited concentrations of B (<20 mg B kg^−1^). Soil used in our experiment had adequate B concentration (ranged from 1.5 to 2.0 mg kg^−1^) to support the crop. The limited mobility of B in cotton may be one reason of why cotton sometimes responds to foliar B application. Dordas (2006) investigated the foliar application of B on cotton and found there was an increase in cotton lint, and concluded that it is possible that the critical levels of B in cotton have been assessed by visual symptoms and not by yield of comparative field studies. It must be noted also that some other studies did not find a positive response to foliar B applications (Heitholt, 1994).

The other mineral that showed an interesting pattern was Ca. The accumulation of Ca in leaves, seeds, and lint was higher in *N* lines than in *F* lines, and this could be explained by the fact that Ca taken up was not used for further physiological and biochemical processing involved in lint fiber development and structure such as the synthesis of cell wall polysaccharides and fatty acids (Gou et al., 2007; Yang et al., 2008; Pang et al., 2010a,b) and the secondary cell wall synthesis (Li et al., 2002, 2005; Wang et al., 2010; Wang and Ruan, 2010). Also, the down-regulation of calcium and phytohormone mediated signals observed at fiber initiation stage in the fuzzless mutants (Padmalatha et al., 2012) could explain the higher levels of Ca in leaves, seeds, and lint due to lower requirements of Ca by fuzzless seed lines. The significant role of Ca in cotton was previously reported and involved in pollen germination (Brewbaker and Kwack, 1963), pollen tube growth (Zhang et al., 1997), stimulation of fertilization (Faure et al., 1994; Tian and Russell, 1997), and egg activation (Digonnet et al., 1997), and this is because Ca is required for vesicle fusion at the tip of the elongating tube (Pierson et al., 1996). It was also found that Ca enhances the antioxidant enzyme activity and protects the plant under oxidative stress conditions through reactive oxygen species (ROS) scavenging (Jiang and Huang, 2001). The apparent explanation that seed Ca in *N* lines was higher than in *F* lines could be due to higher requirements of Ca by *N* lines for physiological and structural functions, may be, for seed protection from severe environmental conditions such as drought and high heat as seeds in *N* lines are covered with little lint compared with *F* lines. If this is the case, then germination rate between *N* and *F* lines should be different as seed of *N* lines may be more hard seeded because of the higher Ca accumulation. The higher Ca content in leaves and lint may support the higher Ca requirement by *N* lines compared with F lines. Because the current results showed higher Ca in leaves and seeds in *N* lines than in *F* lines, it would be worthwhile to further investigate the effects of Ca supply on Ca partitioning in different plant tissues, including seeds and relate that to the rate of ROS enzymes and germination rates.

### Correlations between nutrients in fuzzless and fuzzy near-isogenic lines

In *N* lines, protein was positively correlated with Ca, B, Fe, Mn, and Zn, and oil was positively correlated with C (Table 6). There were positive correlations between Ca and K, Mg, P, B, Fe, Mn, and Zn, and positive correlations between Mg and P, B, Fe, Mn, and Zn. Positive correlations were observed between P and B, Fe, Mn, and Zn, and between S and Mn. Boron was positively correlated with Fe, Mn, and Zn, and Fe had a positive correlation with Mn and Zn. A positive correlation between Mn and Zn was also observed. In *F* lines, positive correlations between protein and B, and between protein and Ca were observed (Table 7). Oil was positively correlated with Zn, and Ca had a positive correlation with B and Mn. Both Mg and P had positive correlation with B, Fe, and Mn. Boron had positive correlations with Fe and Mn, and Fe had positive correlations with Mn and Zn.

**Table 6:** **Pearson correlation coefficient (*R*- and *P*-values) between nutrients in cottonseed of near-isogenic *Gossypium hirsutum* lines expressing the fuzzless/linted seed phenotype across 2012 and 2013**.

		**Protein**	**Oil**	**Ca**	**K**	**Mg**	**P**	**C**	**N**	**S**	**B**	**Fe**	**Mn**	**Zn**
Protein	*R*	1												
	*P*													
Oil	*R*	NS	1											
	*P*													
Ca	*R*	0.63	NS	1										
	*P*	***												
K	*R*	NS	NS	0.36	1									
	*P*			*										
Mg	*R*	NS	NS	0.52	0.54	1								
	*P*			***	***									
P	*R*	NS	NS	0.58	0.61	0.92	1							
	*P*			***	***	***								
C	*R*	NS	0.41	NS	NS	NS	NS	1						
	*P*		**		NS		NS							
N	*R*	NS	NS	NS	NS	NS	NS	NS	1					
	*P*						NS							
S	*R*	NS	NS	NS	NS	NS	NS	NS	NS	1				
	*P*						NS							
B	*R*	0.49	NS	0.70	NS	0.77	0.73	NS	NS	NS	1			
	*P*	**		***		***	***							
Fe	*R*	0.51	NS	0.65	0.40	0.75	0.71	NS	NS	NS	0.74	1		
	*P*	***		***	*	***	***				***			
Mn	*R*	0.40	NS	0.49	NS	0.38	0.35	NS	NS	0.30	0.51	0.45	1	
	*P*	*		**		*	*			*	***	**		
Zn	*R*	0.31	NS	0.65	NS	0.59	0.50	NS	NS	NS	0.75	0.62	0.41	1
	*P*	*		***		***	**				***	***	**	

N = 40;

* p ≤ 0.5;

** p ≤ 0.01;

*** *p ≤ 0.001*.

**Table 7:** **Table 6 Pearson correlation coefficient (*R*- and *P*-values) between nutrients in cottonseed of near-isogenic *Gossypium hirsutum* lines expressing the fuzzy/linted seed phenotype across 2012 and 2013**.

		**Protein**	**Oil**	**Ca**	**K**	**Mg**	**P**	**C**	**N**	**S**	**B**	**Fe**	**Mn**	**Zn**
Protein		1												
Oil	*R*	NS	1											
	*P*													
Ca	*R*	0.41		1										
	*P*	**												
K	*R*	NS	NS	NS	1									
	*P*													
Mg	*R*	NS	NS	NS	NS	1								
	*P*													
P	*R*	NS	NS	NS	NS	0.95	1							
	*P*					***								
C	*R*	NS	NS	NS	NS	NS	NS	1						
	*P*													
N	*R*	NS	NS	NS	NS	NS	NS	NS	1					
	*P*													
S	*R*	NS	NS	NS	NS	NS	NS	NS	NS	1				
	*P*													
B	*R*	0.50	NS	0.41	NS	0.46	0.44	NS	NS	NS	1			
	*P*	***		*		**	**							
Fe	*R*	NS	NS	NS	NS	0.62	0.58	NS	NS	NS	0.54	1		
	*P*					***	***				***			
Mn	*R*	NS	NS	0.36	NS	0.54	0.51	NS	NS	NS	0.61	0.67	1	
	*P*			*		***	***				***	***		
Zn	*R*	NS	0.37	NS	NS	NS	NS	NS	NS	NS	NS	0.31		1
	*P*		*									*		

N = 40;

* p ≤ 0.5;

** p ≤ 0.01;

*** *p ≤ 0.001*.

Correlation between nutrients in *N* lines and *F* lines showed significant positive correlation between some nutrients in both *N* and *F* lines, but some nutrients did not correlate (Tables 6, 7). For example, in *N* lines protein was positively correlated with Ca, B, Fe, Mn, and Zn, suggesting the indirect involvement of these minerals with protein synthesis. Also, the positive correlations between Ca and oil, and between C and oil support the observation of similar trends of these nutrients in *N* lines, and the involvement of carbon in oil metabolism. Nutrients such as P, Ca, and B; Ca, B, and Mn; Mg, B, Fe, and Mn; P, B, and Mn; Fe, Mn, and Zn showed consistency of positive correlation in both *N* and *F* lines. Other nutrients were not consistent between *N* and *F* lines, and this is due to genotypic effects. The positive and negative correlation between cation and anion nutrients were previously reported, although this correlation depends on growth conditions, genotype, and nutrient supply (Mengel and Kirkby, 1982; Marschner, 2012).

**Figure 5:**
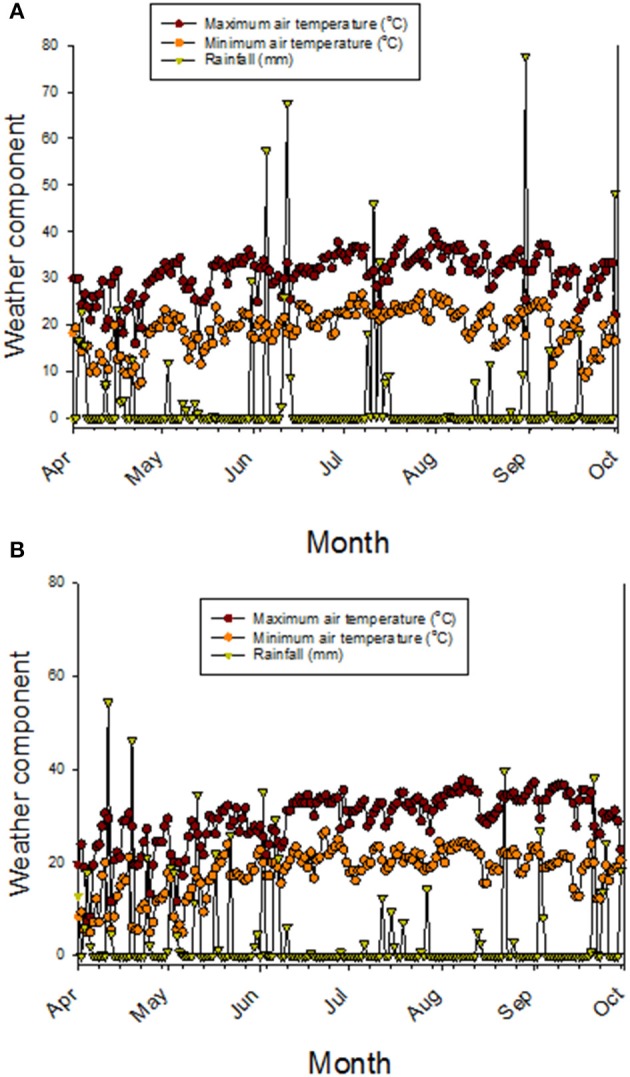
**Maximum and minimum air temperatures, and precipitation (rainfall) in 2012 (A) and in 2013 (B)**. Weather data obtained from MSUCares Stoneville (2015).

Nutrient uptake, translocation, redistribution, and accumulation are processes controlling the accumulation of minerals in seeds (Grusak and DellaPenna, 1999; White and Broadley, 2005), and most of the genetic basis of these process are still not known (Ding et al., 2010). Previous research on fuzzless cottonseed showed that fiber development involves physiological, biochemical, and molecular processes (Turley et al., 2007; Padmalatha et al., 2012). For example, it was found that the fiber development involved processes related to genetics (Turley and Kloth, 2002; Gou et al., 2007; Turley et al., 2007; Zhao et al., 2009; Liu et al., 2012), phytohormones (Yang et al., 2006; Zhang et al., 2011), transcription factors such as MYB25 (Machado et al., 2009), turgor mechanism (Ruan et al., 2003; Wang et al., 2010; Wang and Ruan, 2010), soluble sugars, potassium, and organic acids, ion-transporters (H+-ATPases and K+-transporter) (Wang et al., 2010; Wang and Ruan, 2010); upregulation of potassium and sugar transporters to maintain the turgor pressure (Ruan et al., 2004), carbohydrate and energy metabolism and carbon skeletons for the synthesis of cell wall polysaccharides and fatty acids (Gou et al., 2007; Yang et al., 2008; Pang et al., 2010a,b), and the secondary cell wall synthesis (Li et al., 2002, 2005; Wang et al., 2010; Wang and Ruan, 2010). Because these processes were involved in fiber development, then it is expected to have differences in phenotypic traits such as protein, oil, and minerals between fuzzy and fuzzless cottonseed. This is because of the involvement of carbon skeleton and energy metabolism (Gou et al., 2007; Yang et al., 2008; Pang et al., 2010a,b), K (Ruan et al., 2004; Wang et al., 2010), and Ca (Padmalatha et al., 2012) in fiber development. The only difference between each line set investigated here is cottonseed fuzz trait, so differences in cottonseed composition constituents observed in our study could be explained by the trait as each isoline set has the same genetic background (Bellaloui and Turley, 2013). It must be noted here that information explaining nutrient dynamics in fuzzy and fuzzless cottonseed is almost non-existent (Bellaloui and Turley, 2013), and most of research on fuzzless cottonseed has been on cell biology, genetic, and molecular biology (Padmalatha et al., 2012; Bellaloui and Turley, 2013). However, it is possible that cottonseed differences could be explained by stored energy metabolism differences such as sugars, hormones signaling, and lower lint yields (Turley et al., 2007; Stetina et al., 2014) in fuzzless mutants. These major down regulations, mentioned above, and lower lint yield may have led to a nutrient imbalance, resulting in differences in nutrient uptake, transport, mobility from leaves (source) to seed (sink), and nutrient accumulation in leaves and cottonseed between *N* and *F* isogenic lines during boll development. The weather data (Figures 5A,B) showed that during May-August (reproduction stage period for cotton), the maximum and minimum temperatures in 2012 were higher than in 2013. For example, maximum temperatures in 2012 in May, June, July, August, and September were, respectively, 30.9, 31.7, 33.9, and 33.8°C vs. 26.2, 30.4, 31.5, and 33.8°C in 2013. Rainfall (mm) was higher in April and May in 2013 than in 2012, but in June, July, August, generally the rainfall was higher in 2012 than in 2013. Bearing in mind the rainfall in 1 day can reach upto 77 mm (for example in 2012 in August) or upto 40 mm (for example, in August in 2013). It is clear that the different patterns in temperature and rainfall between years (Figures 5A,B) will impact soil moisture, and consequently nutrient uptake, transport, and accumulation of nutrients in seeds. The effect of rainfall and temperatures in each year could be minimum since the experiment was irrigated and all genotypes were exposed to the same growing conditions. However, we still do not know the response of *F* and *N* genotypes or genotypes in each isoline set (fuzzy or fuzzless) to soil moisture resulted from rainfall or higher temperatures and their effects on nutrients uptake and transport. Therefore, we cannot exclude the effect of weather conditions on seed nutrients accumulation in *F* and *N* genotypes or on genotypes in the two sets of isolines. Further research is needed to determine the response of *F* and *N* genotypes to growing conditions such as drought and heat under field conditions.

It appears that the higher accumulation of nutrients of most *N* lines in their leaves and lower accumulation of nutrients (N, S, and minerals) in seeds of some of the *F* lines could be due to limited mobility of the nutrients from leaves to seed and lower nutrients demand by seeds of these *N* line, resulting in higher accumulation of nutrients in leaves. Some *N* lines did not follow this pattern, and this could be due to genotypic differences and fuzz trait. The real mechanism of how the fuzz development is involved in differential nutrients between *N* and *F* lines are still not understood (Bellaloui and Turley, 2013), and further research on the effects of nutrient supply on nutrient mobility is needed. Also, research is underway, and more biochemical and cellular methods including gene expression profiling based on RNA sequencing will be used to identify the gene responsible for this trait.

## Conclusions

The current research demonstrated that seed protein was higher in fuzzy genotype in all near-isogenic sets, but seed oil was higher in fuzzless genotype in all near-isogenic sets, suggesting possible commercial use for fuzzless seed as source of both protein or oil as the level of protein and oil was competitive with the commercial check. The higher carbon in all fuzzless cottonseed may indicate that higher energy-storage occurred in fuzzless cottonseed. The higher accumulation of most of nutrients in leaves of fuzzless lines and lower accumulation of these nutrients in cottonseed suggested limited mobility and translocation of these nutrients in fuzzless lines compared with fuzzy lines. The research demonstrated that fuzz fiber development altered cottonseed composition, and this may be due to the involvement of fuzz fiber development in carbon and nitrogen metabolism, and the mobility of nutrients from leaves (source) to seed (sink). This information is beneficial to breeders for selection for higher oil or higher protein content, and to physiologist to further understand the mechanisms of mineral movement within plants. Understanding the mechanisms of nutrient mobility from leaves to seed would help for selecting for cottonseed with higher nutritional quality for human consumption and animal feed.

### Conflict of interest statement

The authors declare that the research was conducted in the absence of any commercial or financial relationships that could be construed as a potential conflict of interest.

## References

[B1] Analytical Methods Committee (1959). Analysts. Her majesty's stationery office, London. 84, 214.

[B2] Arpat A. B. Waugh M. Sullivan J. P. Gonzales M. Frisch D. Main D.. (2004). Functional genomics of cell elongation in developing cotton fibers. Plant Mol. Biol. 54, 911–929. 10.1007/s11103-004-0392-y 15604659

[B3] Association of Official Analytical Chemists (AOAC) (1990a). Method 988.05, in Official Methods of Analysis, 15th Edn., ed Helrich K. (Arlington, VA: AOAC), 70.

[B4] Association of Official Analytical Chemists (AOAC) (1990b). Method 920.39, in Official Methods of Analysis, 15th Edn, ed Helrich K. (Arlington, VA: AOAC), 79.

[B5] Bandemer S. L. Schaible P. J. (1944). Determination of iron. A study of the o-phenanthroline method. Ind. Eng. Chem. Anal. Ed. 16, 317–319 10.1021/i560129a013

[B6] Bechere E. Auld D. L. Hequet E. (2009). Development of ‘naked-tufted’ seed coat mutants for potential use in cotton production. Euphytica 167, 333–339 10.1007/s10681-009-9890-y

[B7] Bellaloui N. Gillen A. M. Mengistu A. Kebede H. Fisher D. K. Smith J. R. (2013a). Responses of nitrogen metabolism and seed nutrition to drought stress in soybean genotypes differing in slow-wilting phenotype. Front. Plant Sci. 4:498 10.3389/fpls.2013.00498 PMC3857554 24339829

[B8] Bellaloui N. Hanks J. E. Fisher D. K. Mengistub A. (2009). Soybean seed composition is influenced by within-field variability in soil nutrients. Plant Manag. Netw. 8 10.1094/CM-2009-1203-01-RS

[B9] Bellaloui N. Hu Y. Mengistu A. Kassem M. A. Abel C. A. (2013b). Effects of foliar boron application on seed composition, cell wall boron, and seed δ 15N and δ 13C isotopes in water-stressed soybean plants. Front. Plant Sci. 4:270. 10.3389/fpls.2013.00270 23888163 PMC3719013

[B10] Bellaloui N. Mengistu A. Walker R. R. Young L. D. (2014). Soybean seed composition affected by seeding rates and row spacing in the Midsouth USA. Crop Sci. 54, 1782–1795 10.2135/cropsci2013.07.0463

[B11] Bellaloui N. Smith J. R. Gillen A. M. Ray J. D. (2011). Effects of maturity, genotypic background, and temperature on seed mineral composition in near-isogenic soybean lines in the early soybean production system. Crop Sci. 51, 1161–1171 10.2135/cropsci2010.04.0187

[B12] Bellaloui N. Turley R. B. (2013). Effects of fuzzless cottonseed phenotype on cottonseed nutrient composition in near isogenic cotton (*Gossypium hirsutum* L.) mutant lines under well-watered and water stress conditions. Front. Plant Sci. 4:516. 10.3389/fpls.2013.00516 24416037 PMC3874854

[B13] Boydak E. Alpaslan M. Hayta M. Gercek S. Simsek M. (2002). Seed composition of soybeans grown in the Harran region of Turkey as affected by row spacing and irrigation. J. Agric. Food Chem. 50, 4718–4720. 10.1021/jf0255331 12137503

[B14] Brewbaker J. L. Kwack B. H. (1963). The essential role of calcium ion in pollen germination and pollen tube growth. Am. J. Bot. 50, 859–865 10.2307/2439772

[B15] Brown P. H. Bellaloui N. Wimmer M. A. Bassil E. S. Ruiz J. Hu H. (2002). Boron in plant biology. Plant Biol. 4, 205–223 10.1055/s-2002-25740

[B16] Brown P. H. Shelp B. J. (1997). Boron mobility in plants. Plant Soil 193, 85–101 10.1023/A:1004211925160

[B17] Burton J. W. (1985). Breeding soybean for improved protein quantity and quality, in World Soybean Research Conference III: Proceedings, ed Shibles R. (Ames; Boulder: Westview Press), August 12–17. 361–367.

[B18] Cavell A. J. (1955). The colorimetric determination of phosphorus in plant materials. J. Sci. Food Agric. 6, 479–480. 10.1002/jsfa.274006081425051613

[B19] Digonnet C. Aldon D. Leduc N. Dumas C. Rougier M. (1997). First evidence of a calcium transient in flowering plants at fertilization. Dev. Camb. Engl. 124, 2867–2874. 9247330 10.1242/dev.124.15.2867

[B20] Ding G. Yang M. Hu Y. Liao Y. Shi L. Xu F. (2010). Quantitative trait loci affecting seed mineral concentrations in *Brassica napus* grown with contrasting phosphorus supplies. Ann. Bot. 105, 1221–1234. 10.1093/aob/mcq050 20237116 PMC2887070

[B21] Dordas C. (2006). Foliar boron application affects lint and seed yield and improves seed quality of cotton grown on calcareous soils. Nutr. Cycl. Agroecosyst. 76, 19–28 10.1007/s10705-006-9037-7

[B22] Faure J. E. Digonnet C. Dumas C. (1994). An *in vitro* system for adhesion and fusion of maize gametes. Science 263, 1598–1600. 10.1126/science.263.5153.1598 17744790

[B23] Fletcher R. J. Bell I. P. Lambert J. P. (2004). Public health aspects of food fortification: a question of balance. Proc. Nutr. Soc. 63, 605–614. 10.1079/PNS2004391 15831133

[B24] Gou J. Y. Wang L. J. Chen S. P. Hu W. L. Chen X. Y. (2007). Gene expression and metabolite profiles of cotton fiber during cell elongation and secondary cell wall synthesis. Cell Res. 17, 422–434. 10.1038/sj.cr.7310150 17387330

[B25] Grusak M. A. DellaPenna D. (1999). Improving the nutrient composition of plants to enhance human nutrition and health. Ann. Rev. Plant Physiol. Plant Mol. Biol. 50, 133–161. 10.1146/annurev.arplant.50.1.133 15012206

[B26] Guan X. Lee J. J. Pang M. Shi X. Stelly D. M. Chen Z. J. (2011). Activation of Arabidopsis seed hair development by cotton fiber-related genes. PLoS ONE 6:e21301. 10.1371/journal.pone.0021301 21779324 PMC3136922

[B27] He Z. Shankle M. Zhang H. Way T. R. Tewolde H. Uchimiya M. (2013). Mineral composition of cottonseed is affected by fertilization management practices. Agron. J. 105, 341–350 10.2134/agronj2012.0351

[B28] Heinemann R. J. B. Fagundes P. L. Pinto E. A. Penteado M. V. C. Lanfer-Marquez U. M. (2005). Comparative study of nutrient composition of commercial brown, parboiled and milled rice from Brazil. J. Food Comp. Anal. 18, 287–296 10.1016/j.jfca.2004.07.005

[B29] Heitholt J. J. (1994). Supplemental boron, boll retention percentage, ovary carbohydrates and lint yield in modern cotton genotypes. Agron. J. 86, 492–497 10.2134/agronj1994.00021962008600030007x

[B30] Ji S. J. Lu Y. C. Feng J. X. Wei G. Li J. Shi Y. H.. (2003). Isolation and analyses of genes preferentially expressed during early cotton fiber development by subtractive PCR and cDNA array. Nucleic Acids Res. 31, 2534–2543. 10.1093/nar/gkg358 12736302 PMC156040

[B31] Jiang Y. Huang B. (2001). Effects of calcium on antioxidant activities and water relations associated with heat tolerance in two cool-season grasses. J. Exp. Bot. 52, 341–349. 10.1093/jexbot/52.355.341 11283179

[B32] John M. K. Chuah H. H. Neufeld J. H. (1975). Application of improved azomethine-H method to the determination of boron in soils and plants. Anal. Lett. 8:559–568 10.1080/00032717508058240

[B33] Kebede H. Abbas H. K. Fisher D. K. Bellaloui N. (2013). Relationship between aflatoxin contamination and physiological responses of corn plants under drought and heat stress. Toxins 4, 1385–1403 10.3390/toxins4111385 23202322 PMC3509714

[B34] Li X. B. Cai L. Cheng N. H. Liu J. W. (2002). Molecular characterization of the cotton GhTUB1 gene that is preferentially expressed in fiber. Plant Physiol. 130, 666–674. 10.1104/pp.005538 12376634 PMC166596

[B35] Li X. B. Fan X. P. Wang X. L. Cai L. Yang W. C. (2005). The cotton ACTIN1 gene is functionally expressed in fibers and participates in fiber elongation. Plant Cell 17, 859–875. 10.1105/tpc.104.029629 15722467 PMC1069704

[B36] Liu K. Han M. Zhang C. Yao L. Sun J. Zhang T. (2012). Comparative proteomic analysis reveals the mechanisms governing cotton fiber differentiation and initiation. J. Proteomics 75, 845–856. 10.1016/j.jprot.2011.09.025 22015716

[B37] Loeppert R. L. Inskeep W. P. (1996). Colorimetric determination of ferrous iron and ferric iron by the 1,10-phenanthroline method, in Methods of Soil Analysis: Part 3, Chemical Methods, ed Bigham J. M. (Madison, WI: SSSA), 659–661.

[B38] Lohse G. (1982). Microanalytical azomethine-H method for boron determination in plant tissue. Commun. Soil Sci. Plant Anal. 13, 127–134 10.1080/00103628209367251

[B39] Lu K. Li L. Zheng X. Zhang Z. Mou T. Hu Z. (2008). Quantitative trait loci controlling Cu, Ca, Zn, Mn and Fe content in rice grains. J. Genet. 87, 305–310. 10.1007/s12041-008-0049-8 19147920

[B40] Machado A. Wu Y. Yang Y. Llewellyn D. J. Dennis E. S. (2009). The MYB transcription factor GhMYB25 regulates early fiber and trichome development. Plant J. 59, 52–62. 10.1111/j.1365-313X.2009.03847.x 19309462

[B41] Marschner P. (2012). Marschner's Mineral Nutrition of Higher Plants. 3rd Edn San Diego, CA: Academic Press.

[B42] Mengel K. Kirkby E. A. (1982). Principles of Plant Nutrition, 3rd Edn Worblaufen; Bern: International Potash Institute.

[B42a] MSUCares, Stoneville M. S. (2015). (http://ext.msstate.edu/anr/drec/weather.cgi). Verified on February 10, 2015.

[B43] Padmalatha K. V. Patil D. P. Kumar K. Dhandapani G. Kanakachari M. Phanindra M. L. V.. (2012). Functional genomics of fuzzless-lintless mutant of *Gossypium hirsutum* L. cv. MCU5 reveal key genes and pathways involved in cotton fiber initiation and elongation. BMC Genomics 13:624–638. 10.1186/1471-2164-13-624 23151214 PMC3556503

[B44] Pang C. Y. Wang H. Pang Y. Xu C. Jiao Y. Qin Y. M. X.. (2010a). Comparative proteomics indicates that biosynthesis of pectic precursors is important for cotton fiber and Arabidopsis root hair elongation. Mol. Cell. Proteomics 9, 2019–2033. 10.1074/mcp.M110.000349 20525998 PMC2938120

[B45] Pang C. Y. Wang H. Song W. Q. Zhu Y. X. (2010b). The cotton ATP synthase δ 1 subunit is required to maintain a higher ATP/ADP ratio that facilitates rapid fiber cell elongation. Plant Biol. 12, 903–909. 10.1111/j.1438-8677.2009.00313.x 21040305

[B46] Pettigrew W. T. Dowd M. K. (2011). Varying planting dates or irrigation regimes alters cottonseed composition. Crop Sci. 51, 2155–2164 10.2135/cropsci2011.02.0085

[B47] Pierson E. S. Miller D. D. Callaham D. A. van Aken J. Hackett G. Hepler P. K. (1996). Tip-localized calcium entry fluctuates during pollen tube growth. Dev. Biol. 174, 160–173. 10.1006/dbio.1996.0060 8626016

[B48] Ray J. D. Fritschi F. B. Heatherly L. G. (2006). Large application of fertilizer N at planting affects seed protein and oil concentrations in the early soybean production system. Field Crops Res. 99, 67–74 10.1016/j.fcr.2006.03.006

[B49] Ruan Y. L. Llewellyn D. J. Furbank R. T. (2003). Suppression of sucrose synthase gene expression represses cotton fiber cell initiation, elongation, and seed development. Plant Cell 15, 952–964. 10.1105/tpc.010108 12671090 PMC152341

[B50] Ruan Y. L. Xu S. M. White R. Furbank R. T. (2004). Genotypic and developmental evidence for the role of plasmodesmatal regulation in cotton fiber elongation mediated by callose turnover. Plant Physiol. 136, 4104–4113. 10.1104/pp.104.051540 15557097 PMC535841

[B51] Samman S. Naghii M. R. Lyons Wall P. M. Verus A. P. (1998). The nutritional and metabolic effects of boron in humans and animals. Biol. Trace Elem. Res. 66, 227–235. 10.1007/BF02783140 10050922

[B52] SAS (2002-2012). Statistical Analysis System. (Cary, NC: SAS Institute Inc).

[B53] Seagull R. W. Giavalis S. (2004). Pre- and post- anthesis application of exogenous hormones alters fiber production in *Gossypium hirsutum* L. cultivar Maxxa GTO. J. Cotton Sci. 8, 105–111 Available online at: https://www.cotton.org/journal/2004-08/2/upload/jcs08-105.pdf

[B54] Shi Y. H. Zhu S. W. Mao X. Z. Feng J. X. Qin Y. M. Zhang L.. (2006). Transcriptome profiling, molecular biological, and physiological studies reveal a major role for ethylene in cotton fiber cell elongation. Plant Cell. 18, 651–664. 10.1105/tpc.105.040303 16461577 PMC1383640

[B55] Stetina S. R. Turley R. B. Bellaloui N. Boykin J. C. (2014). Yield and fiber quality of five pairs of near-isogenic cotton (*Gossypium hirsutum* L.) lines expressing fuzzless/linted and fuzzy/linted seed phenotypes. J. Crop Improv. 28, 680–699. 10.1080/15427528.2014.93190124416037

[B56] Stewart J. M. (1975). Fiber initiation on the cotton ovule (*Gossypium hirsutum*). Am. J. Bot. 62, 723–730 10.2307/2442061

[B57] Tian H. Q. Russell S. D. (1997). Micromanipulation of male and female gametes of *Nicotiana tabacum*: II. Preliminary attempts for *in vitro* fertilization and egg cell culture. Plant Cell Rep. 16, 657–661 10.1007/BF01275510 30727614

[B58] Turley R. B. Kloth R. H. (2002). Identification of a third fuzzless seed locus in upland cotton (*Gossypium hirsutum* L.). J. Hered. 93, 359–364. 10.1093/jhered/93.5.359 12547925

[B59] Turley R. B. Vaughn K. C. Scheffler J. A. (2007). Lint development and properties of fifteen fuzzless seed lines of Upland cotton (*Gossypium hirsutum* L.). Euphytica 156, 57–65 10.1007/s10681-006-9351-9

[B60] Walford S. A. Wu Y. Llewellyn D. J. Dennis E. S. (2011). GhMYB25-like: a key factor in early cotton fiber development. Plant J. 65, 785–797. 10.1111/j.1365-313X.2010.04464.x 21235650

[B61] Wang J. Wang H. Y. Zhao P. M. Han L. B. Jiao G. L. Zheng Y. Y.. (2010). Overexpression of a profilin (GhPFN2) promotes the progression of developmental phases in cotton fibers. Plant Cell Physiol. 51, 1276–1290. 10.1093/pcp/pcq086 20558432

[B62] Wang L. Ruan Y. L. (2010). Unraveling mechanisms of cell expansion linking solute transport, metabolism, plasmodesmtal gating and cell wall dynamics. Plant Signal. Behav. 5, 1561–1564. 10.4161/psb.5.12.13568 21139427 PMC3115103

[B63] White P. J. Broadley M. R. (2005). Biofortifying crops with essential mineral elements. Trends Plant Sci. 10, 586–593. 10.1016/j.tplants.2005.10.001 16271501

[B64] Wilcox J. R. Shibles R. M. (2001). Interrelationships among seed quality attributes in soybean. Crop Sci. 41, 11–14 10.2135/cropsci2001.41111x

[B65] Wilkins T. A. Arpat A. B. (2005). The cotton fiber transcriptome. Physiol. Plant. 124, 295–300 10.1111/j.1399-3054.2005.00514.x

[B66] Yang S. S. Cheung F. Lee J. J. Ha M. Wei N. E. Sze S. H.. (2006). Accumulation of genome-specific transcripts, transcription factors and phytohormonal regulators during early stages of fiber cell development in allotetraploid cotton. Plant J. 47, 761–775. 10.1111/j.1365-313X.2006.02829.x 16889650 PMC4367961

[B67] Yang Y. W. Bian S. M. Yao Y. Liu J. Y. (2008). Comparative proteomic analysis provides new insights into the fiber elongating process in cotton. J. Proteome Res. 7, 4623–4637. 10.1021/pr800550q 18823139

[B68] Yu J. Yu S. Fan S. Song M. Zhai H. Li X.. (2012). Mapping quantitative trait loci for cottonseed oil, protein and gossypol content in a *Gossypium hirsutum* × *Gossypium barbadense* backcross inbred line population. Euphytica 187, 191–201. 10.1007/s10681-012-0630-3 23064252

[B69] Zhang J. S. Yang H. Y. Zhu L. Tong H. (1997). Ultracytochemical localization of calcium in the pollen tube track of cotton gynoecium. Acta Bot. Sin. 39, 121–125.

[B70] Zhang M. W. Guo B. J. Peng Z. M. (2004). Genetic effects on Fe, Zn, Mn and P contents in indica black pericarp rice and their genetic correlations with grain characteristics. Euphytica 135, 315–323. 10.1023/B:EUPH.0000013340.98344.6012200859

[B71] Zhang M. Zheng X. Song S. Zeng Q. Hou L. Li D.. (2011). Spatiotemporal manipulation of auxin biosynthesis in cotton ovule epidermal cells enhances fiber yield and quality. Nat. Biotechnol. 29, 453–458. 10.1038/nbt.1843 21478877

[B72] Zhao P. M. Wang L. L. Han L. B. Wang J. Yao Y. Wang H. Y.. (2009). Proteomic identification of differentially expressed proteins in the ligon lintless mutant of upland cotton (*Gossypium hirsutum* L.). J. Proteome Res. 9, 1076–1087. 10.1021/pr900975t 19954254

